# A spatial model to quantify the mortality impact of service delivery in Sub-Saharan Africa: an ecological design utilizing data from South Africa

**DOI:** 10.1186/1476-072X-12-8

**Published:** 2013-02-20

**Authors:** Kurt Sartorius, Benn KD Sartorius

**Affiliations:** 1 School of Accountancy, Faculty of Commerce, Law and Management, University of the Witwatersrand, Johannesburg, South Africa; 2 School of Public Health, Faculty of Health Sciences, University of the Witwatersrand, Johannesburg, South Africa

**Keywords:** Service delivery, Mortality, Poverty, Income inequality, Population density

## Abstract

**Background:**

Sub Saharan Africa is confronted with a wide range of interlinked health and economic problems that include high levels of mortality and poor service delivery. The objective of the paper is to develop a spatial model for Sub-Saharan Africa that can quantify the mortality impact of (poor) service delivery at sub-district level in order to integrate related health and local level policy interventions. In this regard, an expanded composite service delivery index was developed, and the data were analysed using a Bayesian Poisson spatial model.

**Results:**

The results indicate significant differences in the risk of mortality and poor service delivery at sub-district level. In particular, the results indicate clusters of high mortality and poor service delivery in two of the bigger, poorer provinces with large rural communities. Conversely, two of the wealthier provinces have lower levels of mortality and higher levels of service delivery, but income inequality is more widespread. The bivariate and multivariate models, moreover, reflect significant positive linkages (p < 0.01) between increased mortality and poor service delivery after adjusting for HIV/AIDS, income inequality, population density and the protective influence of metropolitan areas. Finally, the hypothesized provision of a basket of services reduced the mortality rate in South Africa’s 248 sub-districts by an average of 5.3 (0.3-15.4) deaths per 1000.

**Conclusion:**

The results indicate that the model can accurately plot mortality and service delivery “hotspots’ at sub-district level, as well as explain their associations and causality. A mortality reduction index shows that mortality in the highest risk sub-districts can be reduced by as much as 15.4 deaths per 1000 by providing a range of basic services. The ability to use the model in a wider SSA context and elsewhere is also feasible given the innovative use of available databases. Finally, the paper illustrates the importance of developing policy in SSA that can simultaneously solve both economic and health problems.

## Background

Sub-Saharan Africa (SSA) has a history of material deprivation that has been widely explained by the pervasive impact of colonization, as well as because of inept and corrupt post-liberation governments [1]. In addition, economic growth in the region has declined as a result of limited foreign investment that has exacerbated problems like poverty and income inequality [2]. As a result of these factors, as well as inefficiency, many of the countries in the region have been unable to provide basic services to their citizens [3,4]. Poorer communities within these countries are often denied basic services like refuse removal, water and sanitation [1,5]. In parallel with the problems of material deprivation, mortality has also increased in recent decades in SSA largely due to the high level of HIV/AIDS infection that has taxed the limited health care resources of the region [6,7].

The relationship between mortality and its social determinants has been widely reflected in the literature [8]. In this regard, poverty and income inequality elevate the risk of disease and mortality because they promote differential access to limited services and facilities like water and sanitation that are often regarded as primary drivers of health [5,9,10]. At household levels, this risk is further elevated if family members are uneducated and unemployed [11]. Poor service delivery, therefore, coupled with other household variables, has direct health and economic consequences (see Conceptual Framework) because a lack of basic services reduces productivity, it increases cost and makes households more susceptible to death and disease [1,12-14].

The governments of this region are, therefore, confronted with health and development problems that can only be resolved across multiple government departments. Given the limited resources of many of the countries in SSA, a need exists to develop integrated policy to simultaneously solve both economic and public health problems. The objective of this paper is to develop a practical model for the region that can spatially link and explain mortality and service delivery at sub-district level. In this regard, the model will first spatially quantify the risk of mortality and poor service delivery at national, regional and sub-district level in order to identify high risk clusters (hotspots). Secondly, the associations between mortality and a composite service delivery index will be tested taking into account confounders like income inequality. Thirdly, the model will develop a mortality reduction index to illustrate the potential mortality impact of providing different categories of service delivery. In this regard, a reduction in mortality is quantified in response to the provision of specific services so that the relevant authorities can evaluate the cost benefits of an integrated intervention. In order to develop the proposed model, the paper develops an expanded composite delivery index. South African census data is then used to illustrate the suggested generic process (see justification and limitations in Methods and Discussion sections).

## Results

The results first quantify the risk of mortality and poor service provision in South Africa at national, provincial and local municipality level in order to identify high risk areas. The results then examine whether there are any statistically significant linkages between these two phenomena and other confounding variables like income inequality and HIV seroprevalence. Finally, the mortality impact of providing basic services is calculated by using its multivariable adjusted coefficient and local municipality prevalence.

### The geography of mortality and poor service provision (South Africa)

**Figure 1:**
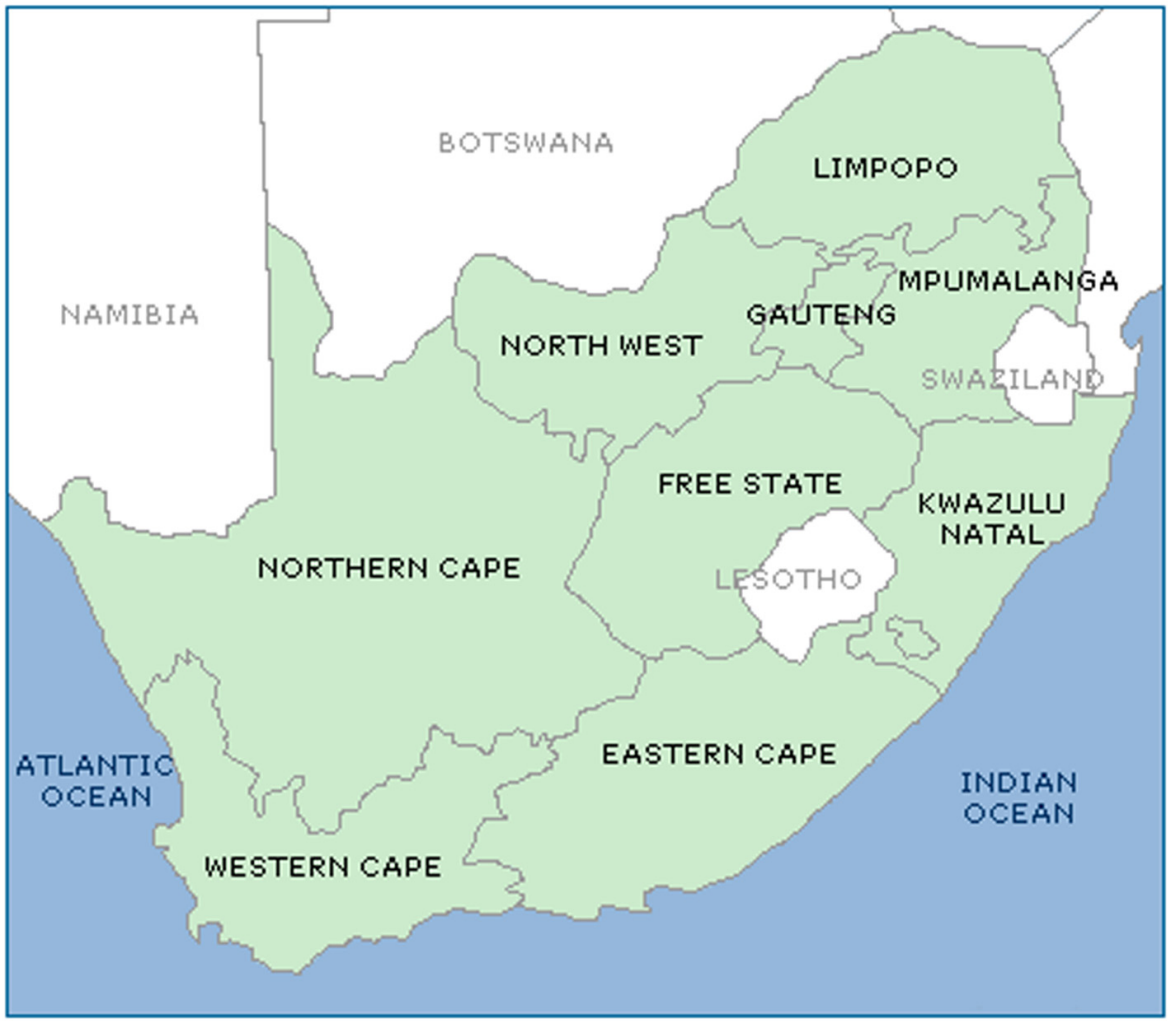
Map of South Africa, with provinces and neighboring countries.

South Africa is administratively divided into nine provinces, illustrated in Figure 1. These provinces consist of 52 districts (46 district municipalities and 6 metropolitans) that can be disaggregated to 248 local municipalities. The highest overall provincial mortality rate was observed in Free State followed by Kwazulu-Natal and Eastern Cape (Table 1), though the difference in mortality rates was not statistically significant when comparing the top four provinces. However we did observe a significantly lower mortality rate in Northwest, Northern Cape and Limpopo compared to the top four and, similarly, a significant reduction in mortality in Gauteng and Western Cape relative to the abovementioned three provinces. The poorest level of service delivery (based on a service non-delivery score) was observed in Limpopo followed by Kwazulu Natal and Eastern Cape. Interestingly, mortality ranks 2–5 and 9 corresponded with the identical rank with regards to service non-delivery (missing data is noted for mortality in Limpopo).

**Table 1:** Mortality and service non-delivery levels and rank at the provincial level, South Africa, 2007

**Province**	**Mortality rate per 1000 (95% CI)**	**Rank (mortality)**	** Service (non) delivery score (95% CI)**	**Rank (service delivery)**
Free State	19.58 (19.42,19.75)	1	64.2 (55.35,73.06)	6
Kwazulu Natal	19.22 (19.13,19.3)	1	113.01 (97.94,128.08)	2
Eastern Cape	19.19 (19.08,19.29)	1	103.18 (78.57,127.79)	3
Mpumalanga	19.14 (19,19.28)	1	98.39 (84.05,112.73)	4
North West	15.31 (15.18,15.45)	5	89.27 (76.31,102.23)	5
Northern Cape	15.15 (14.91,15.38)	5	36.06 (27.61,44.52)	8
Limpopo	11.53 (11.44,11.62)	7	122.69 (104.67,140.7)	1
Gauteng	10.84 (10.78,10.9)	8	48.46 (38.28,58.63)	7
Western Cape	8.02 (7.94,8.1)	9	27.86 (22.35,33.36)	9

**Figure 2:**
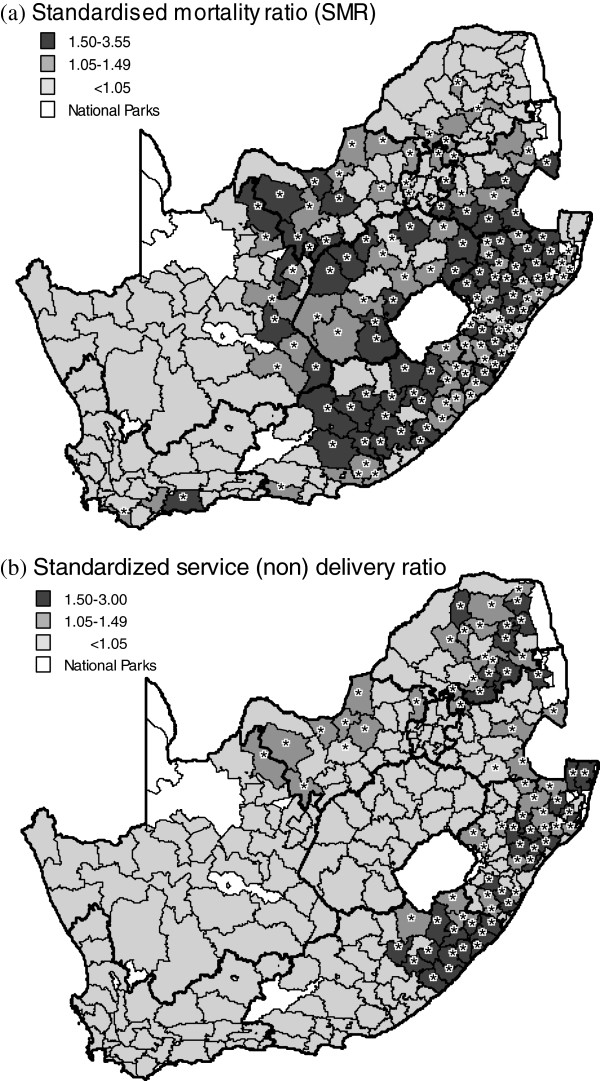
**(a) Chloropleth map plotting the standardised mortality ratio (SMR) and (b) standardised poor service ratio (observed/expected) using a Bayesian BYM convolution Poisson model (baseline model containing only a constant and the conditional autoregressive parameters – see Appendix 1) by local municipality, South Africa, 2007.** Note: areas which significantly exceeded the expected null value (ratio > 1), based on exceedance probabilities, are highlighted with an asterisk; provincial boundaries in bold.

The spatial distribution of mortality and service delivery at local municipality level, based on the Bayesian risk model, is illustrated in Figure 2a and 2b. Local municipalities that significantly exceeded a standardized mortality, or poor service delivery ratio of one, have been indicated with an asterisk. In this regard, similar clusters of high mortality and poor service delivery are indicated in both Kwazulu-Natal and the Eastern Cape. Conversely, Gauteng and the Western Cape reflect higher levels of service delivery and much lower levels of mortality (a single isolated cluster). Lower levels of association in Northwest indicate high levels of mortality are matched with intermediate levels of service delivery. Furthermore, poor levels of service delivery in Limpopo (northeast) are not matched with high levels of mortality but mortality in the province (Limpopo province) has potentially been underestimated due to sampling (and other) errors [15,16]. Little association appears to exist between clusters of high mortality and poor service delivery in the Free State and Mpumalanga. The risk of poor service provision, illustrated in Figure 2b, reflects that the highest aggregate level of poor delivery (standardised ratio >1) was found in Limpopo, followed by the Eastern Cape, Kwazulu-Natal and Mpumalanga. Conversely, the Western Cape demonstrated the highest level of service delivery (far fewer observations with poor delivery compared to expected), followed by the Northern Cape, Gauteng, Free State and North West. Total service delivery in the six main metropolitan areas reveals the highest level of service delivery was delivered in the Cape Town Metropolitan followed by the Johannesburg Metropolitan (Gauteng). Conversely, the lowest level of metropolitan service delivery was demonstrated in the Tshwane (Gauteng) and Ekhuruleni Metropolitan’s (Gauteng).

### Poor service delivery and mortality: the linkages

This section evaluates bivariate associations between mortality, service delivery, income inequality, living within a metropolitan, high population density and HIV seroprevalence before developing a Bayesian multivariable convolution model.

#### Bivariate associations

A highly significant and strong association was observed between increasing mortality and increasingly poor service delivery (Relative risk [RR] = 1.21, p < 0.001) (Table 2). Similarly local municipalities with high income inequality, high population density and increasing HIV seroprevalence were also associated with significantly higher risk. Metropolitan’s areas appeared to have a significantly lower risk compared to non-metropolitan municipalities.

**Table 2:** Bayesian multivariable local municipality mortality risk factor analysis, South Africa, 2007

**Indicator**	**Bivariate analyses (unadjusted)**		**Multivariable model (adjusted)**	
	**Standard Poisson model **^**i**^		**BYM CAR **^**ii **^**spatial Poisson model**	
	**RR (95% CI)**	**p-value**	**RR (95% BCI **^**iii**^**)**	**p-value**
Composite service delivery index score ^iv^	2.90 (1.99,4.20)	<0.001	1.84 (1.43,2.34)	<0.001
High local municipality income inequality	1.23 (1.04,1.46)	0.015	1.14 (1.02,1.29)	0.024
Low-medium district population density	1			
High density ^vi^ – non-metropolitan	1.13 (1.00,1.28)	0.050	0.97 (0.87,1.09)	0.648
High density - metropolitan municipality	0.66 (0.52,0.83)	<0.001	0.73 (0.65,0.82)	<0.001
District antenatal HIV sero-prevalence	1.02 (1.01,1.03)	<0.001	1.02 (1.02,1.03)	<0.001

i: robust standard errors to adjust for local municipality “cluster”.

ii: incorporated an unstructured local municipality random effect and a structured normal CAR spatial random effect.

iii: Bayesian credibility interval.

iv: increasing score indicates increasingly poor service delivery (square transformation used due to violation of linear assumption in Poisson framework).

v: lower tertiale.

vi: upper quartile and adjusting for metropolitan.

#### A multivariable model

The results of a Bayesian multivariable model, illustrated in Table 2, show multivariable adjusted association between mortality and poor service delivery, income inequality, population density, metropolitan residence as well as HIV seroprevalence. We include antenatal HIV seroprevalence in the multivariable model to adjust for its potential confounding influence on local municipality level mortality. The results suggest mortality is still significantly influenced by the level of service provision following multivariable adjustment (RR = 1.83, p < 0.001). We also observe that increasing antenatal HIV sero-prevalence significantly increases the risk of local municipality level mortality. High income equality within the local municipality also remained a significant predictor of increase mortality risk. Residence within a metropolitan area remained significantly protective (RR = 0.73, p < 0.001). High population density in non-metropolitan areas was no longer significant following multivariable adjustment.

### Service delivery provision and mortality reduction at local municipal level

**Figure 3:**
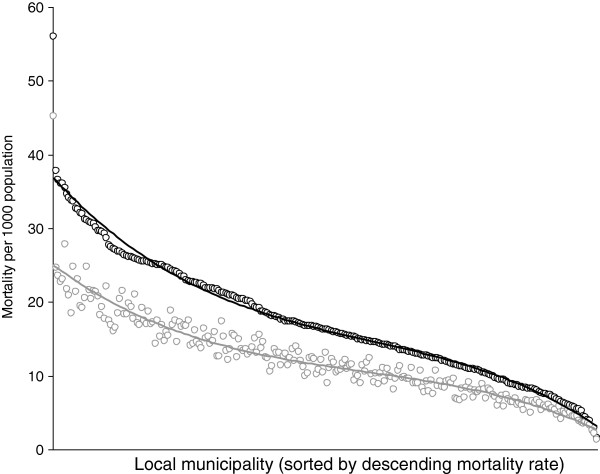
Mortality reduction proportion at local municipality level (quaternary unit): observed (black) and projected reduction (grey) based on the provision of service delivery (removal of attributable factor).

The hypothesized provision of a range of services, illustrated in Figure 3, compares the actual average observed mortality (black curve) of local municipalities with a projected reduction in mortality (grey curve). The projected reduced level of mortality (grey curve) indicates the potential number of deaths per 1000 that could be saved as a result of the provision of a range of services. The attributable fraction estimates suggest that the provision of a range of basic services could result in an overall reduction (black to grey) of 5.3 deaths per 1000 population (95% CI: 4.9-5.7) in South Africa’s 248 local municipalities. Of the 5.3 deaths per 1000 approximately 50 per cent (2.4 deaths per 1000) could be attributed to lack of schooling followed by lack of electricity (0.9) and water provision (0.8). This overall reduction in mortality, ranged between 0.07 and 15.5 deaths per 1000, with the higher risk mortality municipalities (LHS x-axis) reflecting the biggest potential reductions in mortality.

## Discussion

The paper makes a number of contributions to the literature. Firstly, the spatial distribution of excess mortality and service delivery extends the findings of a number of local South African studies. In this regard, the spatial risk of mortality at provincial, district and sub-district levels confirms and extends previous national studies that show higher levels of mortality at provincial level only in the poorer, more rural provinces like the Eastern Cape, Kwazulu-Natal, Northwest, Limpopo and the Free State [16,17]. The limitations of the results regarding the ranking of the four highest mortality provinces (Table 1) are acknowledged, however, as a result of very small mean value differences and overlapping confidence intervals. The increased mortality rate reflected in the study period has been primarily driven by the HIV/AIDS epidemic. After the introduction of anti-retroviral therapy (ART) in 2004, however, the mortality rate from this epidemic appears to have levelled off in 2007 and then declined [18]. Interestingly, the results indicate lower levels of mortality in the metropolitan areas, where income inequality is higher, demonstrating a significant protective effect (e.g. Cape Town Metropolitan).

Furthermore, the spatial distribution of the risk of poor service delivery at sub-district level maps this phenomena for the first time in the region, as well as extends similar studies in South Africa as a result of the development of a service delivery basket that includes variables like education and health facilities [19,20]. Higher levels of service delivery are generally reflected in the wealthier provinces like Gauteng and the Western Cape that generate 48 per cent of the national GDP and reflect higher levels of GDP per capita. Income inequality, however, was higher in the richer provinces with the exception of Northern Cape which only contributes 2.8 per cent of South Africa’s GDP. However, less than three per cent of the country’s population are resident in this province thus enabling it to provide higher levels of service delivery [21,22]. Conversely, poorer provinces like Limpopo and the Eastern Cape generally reflect a lower GDP per capita, lower levels of income inequality and poorer levels of service delivery. Kwazulu-Natal, however, reflects poorer levels of service delivery despite generating over 16 per cent of national GDP, because of the urbanized focus of economic policy, as well as its large rural population outside metropolitan areas [21,22].

Secondly, the paper makes a contribution that can be extended to a wider regional African context. In this regard, the association between mortality and a composite basket of municipal services extends the literature examining the social determinants of mortality by illustrating the important role of local government [8]. An absence of service delivery, and its related impact on mortality at sub-district level, is illustrated in the spatial maps (Figure 2a and 2b) that show similar areas of poor service delivery and high mortality in the local municipalities of Eastern Cape, Kwazulu-Natal, Gauteng and Western Cape. These associations are supported by the bivariate and multivariable models that show significant relationships exist between mortality and poor service provision after adjusting for the confounding influence of HIV/AIDS. The paper, thus, confirms the importance of basic services such as water and sanitation (also refuse removal) and their effect on reducing diarrhoeal and other waterborne diseases [5,8,23]. Poor levels of education, however, compound the problems of household health because of their linkages with high risk behaviour, poor hygiene and unemployment [5]. A shortfall in the results was a lack of correlation between poor service delivery and “low” mortality in Limpopo that may, however, be explained by sampling error and potential underestimation of deaths in this province [24,25].

The results, therefore, provide strong empirical support for the findings of a number of other Sub-Saharan African studies that link mortality with material deprivation as a result of income inequality, poor education, a lack of energy, and poor access to water, refuse removal and healthcare facilities and healthcare facilities [8,13,18,26-31]. In addition, the results also support the association between income inequality and mortality (outside metropolitan areas) because income inequality exacerbates a lack of access to limited health facilities [9].

Thirdly, the paper estimates potential local small area mortality reduction that can be applied in an international context to illustrate the cost benefits of local service provision. In support of this contention, the results indicate that an overall potential reduction of 5.3 deaths per 1000 population could be achieved by providing a composite basket of services that range from the provision of potable water to providing health and education facilities. If the overall number of 5.3 deaths per 1000 is disaggregated, our findings underline the importance of the linkages between mortality and a lack of education [8] by showing that almost 50% of the potential reduction can be attributed to providing education. Furthermore, the results indicate that mortality could potentially be reduced by as much as 15.4 deaths per 1000 population in some of the more extreme local municipality “hotspots”. From a policy perspective, therefore, the results suggest that service delivery interventions at municipality level will yield different cost mortality benefits. The differential reduction in mortality, moreover, could be especially important in poorer SSA countries with limited resources that need to be more effectively targeted. The paper, therefore, indicates that the proposed approach can accurately ‘pinpoint’ mortality and service non-delivery, as well as assess the relationships between them. These contributions and the development of a mortality reduction index can be extended beyond the study area, however, some limitations should be considered [8].

Firstly, the replication of the framework will require the innovative use of databases in the region [32]. Databases, for example, like the DHS combined with national and survey data, offer much potential to improve our knowledge of adult mortality in the region [33]. Secondly, South Africa is different from other SSA countries because it is the only middle income country in the region, as well as because it has it has its own unique service delivery and mortality problems. However, similar patterns of mortality have been projected for the region that indicate a decrease in communicable disease related mortality that is countered by higher levels of lifestyle/non-communicable disease and injury related mortality [34,35].

Limitations and potential sampling errors were also identified when reviewing the survey reports [24,25]. Given the ecological nature of the aggregated secondary data used, caution should be taken when making direct causal inferences as ecological fallacy may potentially bias estimates. However, given that the data were extracted down to the smallest administrative areal unit available (namely local municipality or sub-district), we believe this reduces the potential ecological bias in part. Given the cross-sectional design of the primary study caution should also be exercised when assessing temporal aspects of causality. We also highlight potential limitations of the conditional autoregressive model (CAR) [36] used in this study as it may oversimplify neighbour dynamics e.g. potential for cross-classification as individuals (or households) may access services in neighbouring areas or provinces as opposed to where they stay or their immediate neighbouring municipality (or district). Previous studies have demonstrated how the CAR (1) process fails to capture the spatial process and multiple membership multiple classification (MMMC) models [37,38] may represent a more suitable approach. In our future studies utilizing areal data, we will apply both approaches and select the one which most adequately describes the data and spatial process.

### Recommendations

The results, based on a composite service delivery index, suggest policy interventions need to be coordinated by government central planning centres across government departments like health, education, local government and rural development. With the use of spatial targeting, policy can be spatially differentiated on a provincial, as well as district and local municipality basis to coordinate the prioritization of service provision in resource constrained settings. In particular, provinces with areas of poor service delivery and high mortality at local municipality level, like those in Kwazulu-Natal, Limpopo and the Eastern Cape, should be targeted and prioritized. In terms of a high risk area, therefore, local government would first provide basic services like water, sanitation and refuse removal in a particular local municipality. In parallel, healthcare resources and education interventions would be coordinated at provincial and local level to reduce mortality (e.g. ART for HIV/AIDS patients), as well provide healthcare education in clinics and schools. Finally, development initiatives to increase economic activity in an area must be coordinated with the other suggested prior steps because they are unlikely to succeed without the presence of sufficient (and healthy) working aged individuals.

## Conclusion

The paper investigated and modelled the important mortality implications of multifaceted poor service delivery in South Africa with a view to simultaneously solving common interrelated health and economic problems. This could be reproduced in a broader SSA context and elsewhere. Using South African data as an example, spatial modelling first indicated that the geographic risk of high mortality and poor service provision could be pinpointed at local municipality level. Secondly, the results indicated that mortality could be explained as a function of poor service delivery after adjusting for other confounding variables. Finally, the results illustrated the potential mortality benefit in deaths at a population level and at local municipality level, as a result of improved provision of a composite basket of services. The reliability of the findings for a broader application is well supported by other studies in South Africa, as well as the potential to use other data sources in the wider SSA region.

The linkages between service delivery and mortality provide solid further empirical evidence of this phenomenon in a SSA context. The usefulness of the results is that they indicate that poor service delivery directly affects mortality even when adjusting for the influence of HIV/AIDS. The absence of services like the provision of piped water, sanitation and refuse removal have a long history of disease in Africa. The linkages between mortality and variables like education, income inequality and population density have also been well demonstrated, however, the effect of addressing these variables as a service delivery problem is less well tested.

The proposed approach provides a basis to target and explain areas of high risk mortality at local municipality level in SSA, as well as evaluate a return on investment with respect to interventions. However, the composite basket of services illustrates that multiple local problems have to be addressed to reduce mortality. These problems range from the provision of basic services to the establishment of adequate education and healthcare institutions. Policy intervention, therefore, must be solved across multiple government departments at provincial and local level and mortality data, in this regard, can be used as an additional proxy to gauge the efficiency of local government performance.

The results of this paper suggest the need for further research to test the return on investment (mortality reduction) of service delivery provision in a wider regional context, as well as across the urban rural divide. Furthermore, the impact of major health interventions, like the recent introduction of widespread anti-retroviral therapy against HIV/AIDS, will have a major influence on mortality so similar studies need to reflect a more temporal component to the associations investigated in this paper.

## Methods

### The study area

South Africa has been selected because of its importance in the region, as well as because good quality data are available. South Africa generates over 45% of Sub Saharan Africa’s GDP and, until recently, attracted over 30% of foreign direct investment in Africa [4]. Despite the fact that it is the biggest economy in the region, high levels of income inequality, poverty and mortality co-exist [7,39-41]. South Africa is also an example of a wealthier African country that has grappled with the problem of service delivery at local level. In this regard, service delivery statistics (2002 to 2007) show household access to refuse removal has increased from 58.8% to 60.9%, the percentage of households with no toilets has dropped from 12.6% to 8.3%, and access to piped water rose from 68.8% to 71.8%. However, dissatisfaction with the quality of water has increased, as well as the number of people living in informal settlements. In this regard, provinces like the Eastern Cape and Limpopo (see Figure 1) provide the lowest level of access to services like refuse removal, sanitation and piped water [42] and many municipal districts have been unable to provide access to basic services [18,19,39]. The application of the proposed framework, therefore, is expected to yield similar benefits in other SSA countries although it is acknowledged that service delivery and disease patterns may differ from South Africa.

### The data

The data were from a random national cross sectional survey, conducted by Statistics South Africa in 2007, that included demographic indicators (e.g. age, mortality), household service delivery indicators (e.g. water, sanitation, refuse disposal, electricity), and socio-economic data (e.g. income) [15]. A report documenting the representativeness and quality of the survey as well as potential limitations has been published previously [24,25]. An enumeration area (EA) is defined as the smallest geographical unit into which South Africa is divided for enumeration purposes and consists of between 100 and 250 households. This community survey used all of the 80,787 EA’s countrywide in the sampling frame, of which 1,321 were excluded because they were classified as institutions or recreational areas. The EA’s within each municipality were sorted by land use and human settlement type. The second level of the sampling frame involved the re-listing of the dwelling units that could potentially contain one or more households within the respective EA’s. Ten per cent of the total listed dwellings in each EA were then selected on a random sampling basis [15]. The survey sample included 274 348 dwelling units across the nine provinces with a response rate of 93.9% [24].

The following aggregated (ecological) local municipality level data were extracted from the primary Community Survey 2007 database: total population and total deaths (all age groups) was our outcome; education status of household occupants, household services (access to water, water type and distance to nearest water source; household toilet facilities; household refuse removal, settlement type, electricity supply). A composite service delivery index was developed to indicate the risk of poor service provision (see Appendix 1). Finally, the Gini-coefficient, a commonly used measure of income inequality, was also calculated for each of the local municipalities based on the standard deviation of annual household income within that local municipality.

### Conceptual framework

**Figure 4:**
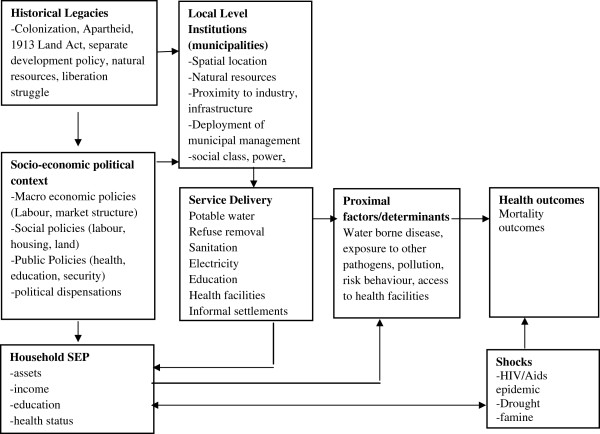
**Service delivery and mortality in South Africa [**[45]**].**

A conceptual framework is presented in Figure 4. Historical legacies in South Africa like colonization and apartheid have influenced the spatial location and economic development of local municipalities with respect to their access to natural resources, infrastructure and management [43]. In the medium term, a combination of these historical legacies, as well as the new political dispensation, continue to influence policy that directs the resources and ability of local government to provide basic services, health facilities and education at household level. In parallel, a combination of these influences have affected household socio economic position (SEP) by influencing family demographics, costs and productivity [39,44]. In recent decades, moreover, shocks like the HIV/AIDS epidemic have also significantly influenced household mortality [6,7]. The risk of mortality in a specific household, in a specific municipality, therefore, is influenced by the availability of a range of basic services, as well as household SEP, because wealthier households have a better ability to handle shocks (like HIV/AIDS) and access alternate facilities. In this regard, poorer households have a higher mortality risk if they have limited access to basic services, because they have fewer resources to cope with injury and disease [8]

### Analysis

The paper employed Bayesian hierarchical modelling to quantify the spatial risk of low service delivery, mortality and income inequality in order to address the problems associated with small area analysis and spatial correlation [46]. Small area studies are more easily interpreted than larger scale studies and are less susceptible to ecological fallacy or bias. Correlation or interdependence of observations in neighbouring or adjoining areas, however, poses a problem because local municipalities in close proximity are often more alike. It is, therefore, important to include the effects of spatial proximity when performing statistical inference on such processes [17]. Bayesian areal (or lattice) models relax the assumption of independence and assume that spatial correlation is influenced by neighbouring locations. These models also allow for prediction or estimation of missed events at un-sampled locations [47]. Bayesian estimators are also widely used in order to obtain reliable estimates for the relative risk when there are sub-areas with small populations and traditional estimates of relative risk lead to unreliable or unstable results [48]. Measurement errors, involving both the numerators and denominators, however, pose a problem in small area studies [49].

The introduction of Markov Chain Monte Carlo (MCMC) methods and software, like WinBUGS, has facilitated the application of Bayesian approaches with respect to the mapping and analysis of many social and health problems. The Besag, York and Molliè [36], or convolution conditional autoregressive (CAR) spatial model, was chosen as it is the most widely used spatial Poisson model for lattice or areal data. This model includes two random-effects terms, namely, a local municipality contiguity (spatial term) and local municipality heterogeneity. This model is more fully explained in Appendix 2. The spatial risk maps, based on a formulation of the above, included no covariates (only a constant and the convolution conditional autoregressive terms). Exceedance probabilities (i.e. smoothed standardised ratio of observed versus expected counts in a given area significantly greater than 1) from the Bayesian spatial modelling approach were used to identify local municipalities with a) significant excess mortality risk (as used previously [17]) and b) significant poor service delivery.

In order to assess the univariate relationships between mortality, service delivery and income inequality, we tested for the degree and significance of correlation between the variables using a classical statistic for correlation, namely, the Pearson correlation coefficient. Correlation significance was assessed at the 5% level. A multivariable Poisson model was then constructed that included mortality, service delivery, income inequality, population density, metropolitans and HIV seroprevalence. We used a multivariable Bayesian spatial Poisson model that was an extension of the BYM convolution model described above (includes random effects for both unstructured and structured heterogeneity) but with covariates included. To calculate expected deaths (E_i_), the overall mortality incidence proportion for 2007 was multiplied by each local municipality’s total population to give the expected number of overall deaths. Given that the service delivery score violated the linear continuous covariate assumption of the Poisson model when compared against the log outcome (deaths), we used a transformed service delivery score (square root) in the uni- and multivariable analyses. We used a more stringent version of Richardson’s criterion [50] to assess covariate significance in which Bayesian exceedance probabilities for a given covariate in excess of 0.95 was deemed to be significant i.e. similar to classical 5% critical cut-off. This is similar to assessing the Bayesian 95% credibility interval (similar interpretation to a classical confidence interval) where the lower 95% bound does not include 1. Further details of the multivariable model are provided in Appendix 3.

We also assessed the degree to which small area units (local municipalities) lack of access to specific types of service delivery (e.g. access to water and sanitation) impacted on mortality. This could provide an indication for policy makers about what intervention(s) to prioritise and the potential reduction in mortality that could be achieved by providing a range of basic services. We also linked risk estimates, associated with the determinants in the multivariable model, with the actual prevalence of exposure to those indicators within the various high risk units identified using the above-mentioned spatial analysis. The following standard formula for calculating an attributable fraction for each determinant was based on its prevalence of exposure (*p*_e_) in a given a real unit, as well as the exponentiated model coefficient (odds ratio [OR]) for that determinant

$$AF_{p}=\frac{p_{e}\left(\mathrm{RR}-1\right)}{1+p_{e}\left(\mathrm{RR}-1\right)}$$

The analysis was carried out in STATA 10.0 SE and WinBUGS. Maps were developed in MapInfo Professional 9.5.

## Appendix 1: Service delivery index

The service (non) delivery index (the higher the score, the poorer the level of service delivery) was a weighted composite score which included the proportion of a local municipality with only a public water supply, no sanitation, no refuse disposal, no electricity and no formal education (schooling). The presence of informal settlements, and the ratio of population to the number of health facilities, was also included in the composite score. Additional data regarding the number of clinics by district were taken from the District Health Barometer for 2007/2008 [51]. Each component was simply weighted to its corresponding normalized z-score ([value-sample mean]/sample standard deviation) before summing the aggregated composite score as some of the individual components are measured on different scales and using this approach ensures replicability across studies. Future studies may consider using differential weighting for individual components based on data quality.

A preliminary correlation matrix, illustrated in Table 3, indicates a significant level of correlation between the components of the service index. The highest correlation is observed between the level of provision of basic services like water, sanitation, refuse disposal, and electricity. To a lesser extent, the provision of basic services correlates with the level of schooling received, as well as the presence of informal settlements and the number of health facilities provided.

**Table 3:** Correlation coefficient matrix of the various individual service delivery components at the local municipality level, South Africa, 2007

**Component**	**Proportion with no water service provider**	**Proportion with no toilet facilities**	**Proportion with no refusal disposal**	**Proportion with no electricity**	**Poor ratio of population size to health facilities**	**Proportion with no schooling**	**Proportion living in informal housing**
Proportion with no water service provider	1.0000	---	---	---	---	---	---
Proportion with no toilet facilities	0.56*	1.0000	---	---	---	---	---
Proportion with no refusal disposal	0.50*	0.65*	1.0000	---	---	---	---
Proportion with no electricity	0.63*	0.61*	0.62*	1.0000	---	---	---
Poor ratio of population size to health facilities	0.18*	0.10	0.16*	0.13*	1.0000	---	---
Proportion with no schooling	0.46*	0.48*	0.45*	0.65*	0.09	1.0000	---
Proportion living in informal housing	−0.37*	−0.25*	−0.26*	−0.34*	0.06	−0.13*	1.0000

*: significant at the 5% level.

## Appendix 2: Bayesian spatial modelling

The Besag, York and Molliè (BYM) [36] or convolution conditional autoregressive (CAR) model is formulated as follows:

$$O_{i}\sim \mathrm{Poisson}\left(E_{i}\lambda_{i}\right)$$

$$\log\left(\lambda_{i}\right)=\alpha +\varepsilon_{i}+\varphi_{i}$$

where λi is the relative risk in area i (local municipality), O_i_ is the number of deaths in area i, E_i_ are the expected number of deaths, ε_i_ is the local municipality heterogeneity term and φ_i_ is the conditional autoregressive (CAR) spatial term. The spatially correlated random effect of the ith region (φ_i_) is based on the sum of the weighted neighbourhood values. We used an adjacency matrix of common boundaries (neighbours) of a given local municipality when modeling this parameter. The unstructured local municipality level random effect was modelled as independent normal distribution ε_i_ ~ N (0,σ^2^_ε_) with variance σ^2^_ε_. Non-informative gamma priors were used for both variance parameters. Besag et al. suggest that the convolution model is more flexible than using a CAR random effect only, since it allows one to quantify how much of the residual disease risk is due to spatially structured variation versus unstructured heterogeneity [36]. The models were fitted using Markov chain Monte Carlo simulation [46]. Deviance Information Criterion (DIC) [52] were used as the criterion for the assessment of the goodness of fit (a lower DIC suggests a better model fit) and, therefore, was used for model selection. The results of the BYM model were plotted on local municipality maps that depicted smoothed standardised mortality ratio estimates and the posterior exceedance probability that these ratios were significantly >1 in a given local municipality. We used a more stringent version of Richardson’s criterion [50] in which probabilities in excess of 0.9 (Richardson’s standard criterion is 0.8) were deemed to be significant in the spatial analysis.

## Appendix 3: Multivariable analysis

The Bayesian multivariable BYM model was simply an extension of the convolution model discussed above which included covariates:

$$\log\left(\mu_{i}\right)=\log\;\left(E_{i}\right)+\alpha +\varepsilon_{i}+\varphi_{i}+\mathrm{Xi}\;\beta$$

where Xi is the vector of covariates and β is the vector of regression coefficients.

Vague Normal distributions were used for β. Coefficients for indicators were exponentiated to represent incidence risk ratios (IRR). Two-chain Markov chain Monte Carlo simulation was used for parameter estimation. Model convergence was assessed by visual inspection of the parameter series plots, and Gelman-Rubin statistics [53]. The final posterior samples obtained after convergence were run until the Monte Carlo error for each parameter was less than 5% of the sample standard deviation. The chains were then sampled until a sample size of 10,000 iterations had been attained.

## Competing interest

The authors declare that they have no competing interests.

## Authors’ contributions

KS literature review and investigated linkages between service delivery and mortality, developed the conceptual framework, drafted the manuscript. BS developed method, data management and processing, data analysis, and assisted with drafting the manuscript (particularly the methods and results). All authors read and approved the final manuscript.
